# Research translation to inform national health policies: learning from multiple perspectives in Uganda

**DOI:** 10.1186/1472-698X-11-S1-S13

**Published:** 2011-03-09

**Authors:** Freddie Ssengooba, Lynn Atuyambe, Suzanne N Kiwanuka, Prasanthi Puvanachandra, Nancy Glass, Adnan A Hyder

**Affiliations:** 1 Makerere University College of Health Sciences, School of Public Health, Kampala, Uganda; 2 Health Systems Program, Department of International Health, Johns Hopkins Bloomberg School of Public Health, Baltimore, MD 21205, USA; 3 Johns Hopkins University, School of Nursing, Baltimore, MD. 21205, USA

## Abstract

**Background:**

Research and evidence can have an impact on policy and practice, resulting in positive outcomes. However, research translation is a complex, dynamic and non-linear process. Although universities in Africa play a major role in generating research evidence, their strategic approaches to influence health policies and decision making are weak. This study was conducted with the aim of understanding the process of translating research into policy in order to guide the strategic direction of Makerere University College of Health Sciences (MakCHS) and similar institutions in their quest to influence health outcomes nationally and globally.

**Methods:**

A case study approach using 30 in-depth interviews with stakeholders involved in two HIV prevention research project was purposively selected. The study sought to analyze the research-to-policy discourses for the prevention of mother-to-child transmission (PMTCT) and safe male circumcision (SMC). The analysis sought to identify entry points, strengths and challenges for research-to-policy processes by interviewing three major groups of stakeholders in Uganda – researchers (8), policy makers (12) and media practitioners (12).

**Results:**

Among the factors that facilitated PMTCT policy uptake and continued implementation were: shared platforms for learning and decision making among stakeholders, implementation pilots to assess feasibility of intervention, the emerging of agencies to undertake operations research and the high visibility of policy benefits to child survival. In contrast, SMC policy processes were stalled for over two years after the findings of the Uganda study was made public. Among other factors, policy makers demanded additional research to assess implementation feasibility of SMC within ordinary health system context. High level leaders also publicly contested the SMC evidence and the underlying values and messages – a situation that reduced the coalition of policy champions.

**Conclusions:**

This study shows that effective translation of PMTCT and SMC research results demanded a “360 degree” approach to assembling additional evidence to inform the implementation feasibility for these two HIV prevention interventions. MakCHS and similar institutions should prioritize implementation research to guide the policy processes about the feasibility of implementing new and effective innovations (e.g. PMTCT or SMC) at a large scale in contexts that may be different from the research environments.

## Background

Uganda has a heavy burden of disease as reflected in poor health indicators. For example, the under five mortality was 137 deaths per 1,000 live births in the years 2001-2005, and the maternal mortality ratio was 435 deaths per 100,000 live births in 2005 [1]. Even though there are some positive trends in several health outcomes, Uganda is not on track to meet Millennium Development Goals (MDGs) 4, 5 and 6. The Government of Uganda is committed to achieving the MDGs, which are reflected in National Development Plan, National Health Policy 2010 to 2020, and Health Sector Strategic and Investment Plan (HSSIP). The HSSIP calls for universal delivery of the Uganda National Minimum Health Care Package captured under the four clusters namely: environmental health, health promotion, disease control and community health initiatives; maternal and child health; prevention, management and control of communicable diseases; and prevention, management and control of non-communicable diseases [2].

A vital entry point to meeting the MDGs is to focus on health systems performance. Effective health systems research and the translation of research into actions are central to enhancing this performance. However, what remains unknown is which strategies actually work in translating such research into policy. Many national health systems (including Uganda) are at a critical juncture – the reform and re-orientation of these health systems requires innovative thinking and a concerted effort to use existing evidence. However, a large proportion of health systems research to-date is unfortunately separated from decision making processes [3,4]. Globally, for knowledge generated from research to be beneficial it should be shared, communicated and translated into policy, practice and/or action [5]. The World Health Organization (WHO) defines *knowledge translation* as “the synthesis, exchange and application of knowledge by relevant stakeholders to accelerate the benefits of global and local innovation in strengthening health systems and improving people’s health” [6]. Research and evidence can have an impact on policy and practice, resulting in positive outcomes. However, knowledge translation is a complex, dynamic and non-linear process that is generally slow. Slow knowledge translation can result in inappropriate decision-making and care to the population [5,7]. Strategies and processes to increase the use of research in decision making are poorly understood and require creativity, intellectual rigor, skills and organizational savvy and endurance [7]. A study by Lavis found that the research-to-policy gaps were related to a myriad of factors that policymakers have to consider, such as the value attached to research evidence in decision making, and to weak relevance of the research evidence and the concerns of policy makers at a point in time [8].

The translation of evidence to policy and improved practice in low and middle income countries (LMIC) has the potential to reduce the gap in morbidity and mortality for disadvantaged populations. For example, access to and utilization of treatments and technology that improves diagnostic accuracy can enhance interventions for rural and urban poor [9,5]. A recent descriptive study on knowledge translation policies and activities of national and international health research funders working in LMIC indicated that there was increasing interest by agencies to engage in knowledge translation activities. However, barriers such as a lack of a common terminology in knowledge translation, limited consideration of which evidence needs translation, and how to best tailor the evidence and the approaches to reach different stakeholders existed. In Africa, it is particularly vital that evidence of effective strategies yielded through research is used to inform policy so that the limited resources available can be put to good use. However, little documentation exists as to what extent this is being done in African countries like Uganda and what processes might facilitate or hinder the bridging of this evidence to policy gap [9-11].

Successful translation of research evidence to policies and programs has taken place in Uganda in the last 5 to10 years. Most of these have arisen from research undertaken in Uganda. For example, the Ministry of Health adopted artemisinin-based combination therapy in the treatment of malaria after studies conducted within the country showed marked resistance to the regimen usually recommended by the Ministry [12-15]. Many research activities in which Uganda participated have also led to global level policy and practice recommendations. Some of the examples include the antiretroviral therapy http://www.medterms.com/script/main/art.asp?articlekey=14370 (ART), preventing mother to child transmission (PMTCT) [16,17] and safe male circumcision in HIV prevention [18]. Integrated management of childhood illness and home-based management of fevers, and Tuberculosis - directly observed treatment strategies have related policies which were adopted by WHO after successful pilot testing in countries including Uganda [19]. In a yet another recent example, routine counseling and testing was quickly adopted as policy to in-patient admissions after research demonstrated overwhelming benefits for prevention and treatment of HIV [20,21].

Overall, this study was conducted with the aim of understanding the process of translating research into policy in order to improve health outcomes related to national health priorities in Uganda. The first specific aim of this study was to learn from the national processes for translating research for PMTCT and safe male circumcision (SMC). Both research projects were conducted in Uganda and at the time of this study, PMTCT had shown a relatively quicker translation into policy and programs compared to SMC. Although SMC research results had been disseminated since 2007, there was little progress in the policy processes by 2009 when this study was launched. The second specific aim of this study was to explore strategies for academic institutions like Makerere University College of Health Sciences (MakCHS) to influence the translation of research into policy and practice. This is a prerequisite for such institutions to influence health outcomes. Given the Ugandan context, two research questions were addressed: 1) Why and how did PMTCT and SMC differ in terms of research-to-policy translation? and 2) What values, interests and criteria are used by key stakeholders in sharing, amplifying and using research evidence? The analysis sought to identify the entry points, strengths, and challenges for advancing the use of evidence into policy development processes in the health sector of Uganda. Such evidence is critical for health training institutions like MakCHS to adopt effective strategies to influence national health development [22].

## Methods

This study was informed by a number of frameworks linking the research-policy interface. One such conceptual framework describes key elements as: processes of research generation and decision-making; the stakeholders; the products; the mediators; and the context [23]. Mediators, individuals or institutions who foster linkages between different stakeholders are described as the most crucial component of the framework that encourages strong research-policy linkages [23]. More recent efforts at framework construction have focused on country-level assessment of linkages between research and action [8]. The proposed framework has four elements: 1) general climate; 2) research production; 3) a mix of push and pull factors; and 4) evaluation approaches. The critical role of a wide range of stakeholders, such as researchers, policymakers, funders and consumers and advocacy groups is also acknowledged in linking research to action. Another recent framework exploring health systems research and its influence on policy processes in low-income countries articulates four “streams of influence” on the research-policy interface: development contexts; stakeholders; accountabilities; and processes [24]. For instance the development context encourages the examination of capabilities of the health system to support priority research as well as acting on evidence when available. In developing country context the external stakeholders – experts and donors may form part of the influence network. In this paper we used the range of variables espoused in these frameworks to help frame the analysis around the major influences of research on health policy in Uganda.

We employed two case studies to draw lessons on how to research influences policy developments in Uganda (Table 1). The PMTCT and SMC were selected based on a consultation workshop in early 2009 in Kampala in which researchers, policy makers and donors participated. A comparison of both cases demonstrates the similarities and differences around HIV prevention in Uganda (Table 1). For both these interventions, a national level network of stakeholders – researchers, policymakers and the media - were identified as critical to the generation, utilization and amplification of evidence respectively. These three categories of stakeholders form the groups that were interviewed in this study. Although the general public is a vital stakeholder in the research-to-policy development, time and logistical constraints did not allow us to pursue this stakeholder group.

**Table 1:** Comparison of case studies-PMTCT and SMC–in Uganda

Concepts	PMTCT	SMC
Status of policy	PMTCT policy adopted in 2001	SMC policy formulation in progress (2009)
Stage in R-2-P processes in 2009	Policy implementation stage	Analysis of policy feasibility and agenda setting
Type of research evidence generated	Implementation level evidence of effectiveness	Proof of concept for SMC – multi-country clinical trials (Rakia, Kisumu and Orange Farm)
Methods used for generating evidence	Large cohorts of program beneficiaries, i.e. children and mothers enrolled in PMTCT programs	Multi-country randomized clinical trial; country level acceptability surveys; service availability services
Objectives of the researchers’ policy engagement	To improve the national policy implementation approaches; Changes to cost-effective approaches	To establish global policy guidelines; establish national SMC programs; mobilize funds for SMC programs
Influential decision-making audiences	National technical level decision makers (MOH WHO, UNAIDS, UNICEF and EGPAF); Makerere College of Health Sciences	Mostly global multilateral agencies e.g. WHO, UNAIDS, Gates Foundation and NIH; MOH and political leaders i.e. president’s opinion about SMC
Secondary audiences	Implementers of PMTCT programs; Funding agencies of PMCTC programs; WHO and UNAIDS (validation of their guidelines)	National level leaders, technical decision makers, media practitioners; general public; HIV funding agencies; Implementers (e.g. hospital managers and surgeons)
Methods for engaging national level decision makers	Researchers are integrated into decision-making fora e.g. PMTCT National Advisory Committee and committees	Transactional or “arms-length” engagement methods by researchers e.g. occasional dissemination events, policy briefings and mass media.

This was a qualitative study where in-depth interviews (IDI) were conducted with key stakeholders including policy makers, technical officers, funders, researchers, and print and media journalists (television, print and radio). The purpose of the in-depth interviews was to provide an understanding of the research to policy process, with a specific focus on PMTCT and SMC. We conducted 30 in-depth interviews with researchers (8), policy makers (12), and media journalists (10). The main selection criteria for researchers and policy makers was their involvement in the decision making around the PMTCT and or SMC process at any time since the year 2000. For the media, they were selected mainly because they were involved in health related reporting on a regular basis in Uganda.

A purposeful sampling frame for the in-depth interviews was constructed in consultation with peers in the PMTCT and SMC domain as well as those who have worked in health related policies in Uganda. First, brainstorming discussions were conducted with a reference group familiar with policy research to generate the initial sampling frame. Then, during interviews, *snowball sampling strategy* was applied and after each in-depth interview was conducted, the interviewee was requested to identify one to two other possible respondents that they deemed relevant to the study. The research team then updated the list of interviewees from which the respondents were drawn.

Interviews were conducted in English and audio recorded (with consent) and transcribed by the research team thereafter. In a few cases, individual were not willing to be audio recorded. In such circumstances, we took hand written notes of the interviews which were later expanded. During data collection phase de-briefing meetings were held with research team members at the end of each day to ensure good quality data and share new and emerging issues. All interviewers were trained in qualitative interview techniques. The interview tool was pilot tested with volunteer colleagues within MakCHS. Transcription was completed within 48 hours following interview.

The initial step for analysis was to read through all the interview transcripts several times while making notes in the transcript. All investigators participated in this process. Analysis was a mixture of Manifest and Latent content analysis techniques. At first manifest content analysis was done in reference to the study conceptual framework. This type of analysis technique allows one to explore what the text says, deals with the content aspect and describes the visible, obvious components. For a closer look of underlying meanings (Latent) content analysis was done and hidden meaning of text was brought forth [25]. Data was therefore condensed without losing quality. Open coding was done and codes were categorized and then themes identified as stipulated by Graneheim and Lundman [25].

This study was approved by the Institutional Review Boards (Ethics Committees) at Makerere University-School of Public Health, Uganda and Johns Hopkins Bloomberg School of Public Health, USA. Permission to conduct the study was also granted by the Uganda National Council of Science and Technology. Verbal consent was solicited from each individual identified as a respondent for the in-depth interview prior to starting the interview.

## Results

This study was conducted among thirty stakeholders (8 researchers, 12 policy makers, 10 media journalists). The respondents were experienced in their work and held influential positions (Table 2). In addition, they had been in their positions of work for a period not less than 5 years. Majority of researchers had done clinical trials, got funding from UN and multilateral agencies, and their primary audiences were the funding agencies. The majority of media practitioners were news editors with more than 5 years of experience. The data from these interviews was collated under three domains of interest; ‘Lessons learnt from PMTCT’, ‘Lessons Learnt from SMC’ and ‘Evidence that Drives Policy’. There are several themes (categories) and sub-themes that emerged under each domain based on content analysis (Table 3).

**Table 2:** Characteristics of the study respondents in Uganda (n=30)

Type of research	Type of policy makers	Type of media
Health Policy	Donors	Print
Epidemiology	Politicians	Radio
Case studies	Technical/Program Manager *	Television *
Clinical Trials*		

*denotes majority of respondents’ expertise

**Table 3:** Example of the content analysis process for the study

Codes	Categories	Theme
-PMTCT National Advisory Committee was chaired by the scientist that carried out the Niverapine study -The PMTCT National Advisory Committee was the main decision-making body -The first author was also a member of this PMTCT National Advisory Committee -Both implementers and research agencies benefited from sharing the decision space	**Shared platforms for learning and decision making**	**Lessons learnt from PMTCT**

-The PMTCT National Advisory Committee commissioned a pilot study to learn about the operations feasibility -A national pilot was established to examine the feasibility of implementing PMTCT -Pilots aimed to assess how integration of PMTCT into ANC might affect the acceptability	**Pilots to assess feasibility of Interventions**	

-History of shared decision-making platform -Sustained collaboration with research agencies like MUJHU, JCRC and PIDC. -Integration of technocrats, basic and operations research scientists along with funding agencies interested -PMTCT National Advisory Committee used research to quickly adjust the guidelines to stop single dose Niverapine when it failed	**Evolution of Agencies to undertake Operational research**	

-700,000 pregnant women are screened annually -Many babies remained HIV negative -The faces of these babies and smiles of grateful mothers are common -Beneficiaries often in media reports -It is harder now to find HIV positive babies -When people see this change they are more willing to implement the policy	**Visibility of the Benefits of PMTCT**	

-SMC policy audiences were primarily global, the nature of policy decision had tight connections to global agencies like WHO, UNAIDS and NIH. -SMC research in Uganda, Kenya and South Africa was funded by global stakeholders -WHO called us, we shared our results with other experts	**Global vis-à-vis National Policy Process**	**Lessons learnt from SMC**

-The demand for a series of addition research evidence -MoH discuss translation of SMC evidence into policy -Feasibility questions emerged -We are also looking at cultural sensitivity	**Demand for feasibility research**	

-SMC benefits happen when a large number has undertaken the service -Benefits are difficult to visualize by the policymakers -Researchers envision complexity of this process -Can existing services to shoulder circumcision	**Less visible evidence for SMC**	

-Sharp differences in values prevail among researchers, policy makers & media -Contribution to science, career development… member of big scientific network -Driven by the need to find simpler and cost-effective solution -Motivated by the duty to inform the public -Maintain the interest of the audience	**Incentives and values**	**Evidence that drives policy**

The main rationale for researcher-policy maker communication was: -PM driven by the need to share positive findings from research -Researchers’ attitudes were not favorable to active engagement and dissemination -Delays in dissemination process “dancing in the corridors” -Media prefer evidence from a locally recognized expert	**Communication among groups**	

-Evidence judged as useful for decision making -We want randomized controlled trials -Decision makers assigned more weight to research that addresses operational problems -Emphasizes the number of the population affected	**Strengths of evidence**	

### Lessons from PMTCT program

The PMTCT policy was adopted in 2001 in Uganda after a pilot study covering three districts in 2000. By the time of this research, PMTCT policy guidelines had undergone several revisions based on the evidence arising from several research agencies that were implementing the program, as well as research around operational challenges (Table 1). Factors that stand out as facilitating the PMTCT policy uptake and continued use of implementation research to improve programs are grouped under the following categories below: ‘shared platforms for learning and decision making’, ‘pilot to assess feasibility of intervention’, ‘evolution of agencies to undertake operations research’ and ‘ visibility of the benefits’.

#### Shared platform for learning and decision making

Under the leadership of UNICEF and the Ministry of Health (MOH), the research scientists in Uganda together with the in-country officials of international agencies like Elizabeth Glaser Pediatric AIDS Foundation, United states Agency for International Development, UNAIDS and World Health Organization formed a PMTCT National Advisory Committee to oversee the national implementation of the findings from the Niverapine study. The PMTCT National Advisory Committee was chaired by the principle research scientist that carried out the Niverapine study and was the main decision making body for developing policy guidelines. One of the authors was also a member of this PMTCT National Advisory Committee and provided expertise for costing and cost-effectiveness of the pilot intervention. Both implementers and research agencies benefited from sharing the decision space (PMTCT committee) and learning together with their colleagues from financing agencies. This enabled funds to be allocated to both implementation research and development of PMTCT programs. As an example one policy maker remarked that:

“When a study is presented here, we subject the findings to the PMTCT National Advisory Committee which has many researchers from academia, partners [donors] and our team [Ministry of Health]. For example, in November 2008, JCRC presented their research finding. The PMTCT advisory committee took up that finding. In January 2009 we wrote a circular to all the implementing facilities to stop using single doses Niverapine.” (Policy Maker-MOH)

#### Pilot to assess feasibility of the intervention

Respondents revealed that early on, the PMTCT National Advisory Committee commissioned a pilot study in three districts to learn about the operational issues and feasibility of implementing the intervention. With support of UNICEF, the feasibility of implementing PMTCT within the antenatal care clinics (ANC), consequent acceptability of PMTCT and ANC service utilizations were studied [26,27]. As expressed by the MOH official, the PMTCT policy and guidelines were adopted following favorable pilot results:

“… the Ministry of health and government said can we take this [PMTCT] up? So, in 2000 and before the program was rolled out [...] pilot studies were conducted in 3 districts and 5 health facilities. The aim was to see how best to roll out PMTCT. Do we need to have specific clinics for PMTCT? Can we integrate it into the existing workforce? Will stigma of HIV testing chase away the mothers? So they did a pilot for 1 year in 2000 and showed that we can integrate the PMTCT program into the existing services. This never scared the mothers from antenatal services. So with that information in mind, the first PMTCT policy was written in 2001” (Policy Maker-MOH).

#### Evolution of agencies to undertake operations research

Data also revealed that given the history of a shared decision-making platform (PMTCT National Advisory Committee) the PMTCT program had a sustained collaboration with research institutions like Makerere University-Johns Hopkins University program, Joint Clinical Research Center, and Paediatric Infectious Disease Clinic. These agencies specialized in PMTCT operations research and had large cohorts of program beneficiaries that made the generation of operational evidence easy and credible. The integration of technocrats, basic and operational research scientists, along with funding agencies interested in PMTCT programs made sharing of implementation research evidence and decision-making more efficient. For example, when operations research showed that mothers of babies exposed at birth to single dose Niverapine were at increased risk developing resistance to the drug, the PMTCT National Advisory Committee quickly adjusted the guidelines to stop single dose Niverapine for PMTCT.

The emergency of specialized PMTCT operations research agencies also helped to reduce the gap between the knowledge demand for policy implementation and the evidence generated from research activities.

“Our organization has supported us to attend WHO meetings as technical advisors, also many of us here [researchers] participate on the PMTCT National Advisory Committees, the national antiretroviral treatment committees and national pediatric and adult guideline committee” (Research Scientist)

In the same vein, another respondent echoed that:

*“Currently we are doing a study to determine if six weeks versus six months of infant prophylaxis with Nevirapine have any difference in benefits […] because we know that if it takes six weeks, infant Nevirapine is easier to implement than the six months.”(Research Scientist)*

#### Visibility of the benefits from PMTCT program

Our interviews revealed that there was high visibility of PMTCT program and its indicators were well known. For example, indicators demonstrating that over 700,000 pregnant women are screened annually and over 80 percent of those found positive were getting enrolled into PMTCT programs [28]. Research programs with cohorts of PMTCT beneficiaries were mentioned to demonstrate that many babies remained HIV negative, and the faces of these babies and smiles of grateful mothers are common in the media reports. A typical quote from a researcher demonstrates this view:

*“… it is harder now to find HIV positive babies because we have shown that PMTCT has reduced the number of babies who get infected. Maybe not as big as we would want*, *but there is truly a change. When people see this change they are more willing to implement the policy*, *strengthen it and invest resources.” (Research Scientist,)*

This view was in agreement with policy makers who affirmed that the contribution of PMTCT results and the positive outcomes are tools for advocacy in resource mobilization.

“We know that PMTCT saves the child from getting HIV and therefore saves the future generation. We have used this to advocate for the PMTCT program and the stakeholders get excited about that. […]One child saved from the program is worth the costs of the program.” (Policy maker)

Despite the high benefits of PMTCT, major implementation challenges limit the scale-up of the programs beyond the 50 percent coverage. Some elements of the program remain unimplemented due to constraints in the health and community systems. These challenges have attracted more implementation research to fine tune the policy and practices. From this perspective some respondents suggested that getting to a policy is not sufficient to guarantee its benefits to the communities or among beneficiaries:

”it would not be straightforward that you will get a program scale-up once there is a policy. We have had the policy for PMTCT but little program scale up. Why has it not moved? Because there are also challenges in implementation that need implementation research.” (Research Scientist-)

### Lessons from SMC program

In contrast to PMTCT that took one year to become national policy, SMC policy processes were in an infantile stage at the time of this research despite two years of vigorous national and international dissemination of the SMC research. The major evidence driving the SMC policy process was from a unique research setting but with the strongest impact due to rigorous methodology and multi-site concordance of results [18]. SMC research scientists and policy makers interviewed in this study showed that the progresses of policy translation were much slower. In this domain analysis indicated that three major drivers of the policy process could be identified under the following categories: ‘global vis-à-vis national policy process’, ‘demand for feasibility research’ and ‘less visible evidence from SMC’.

#### Global vis-à-vis national policy processes

For SMC, targeted policy audiences were primarily global agencies that influence HIV care and prevention policies around the world. Although Ugandan stakeholders like MOH and media were engaged during the research processes, the nature of policy decision and especially financial allocation for SMC and HIV programs had tight connections to, and sought validation from, normative global agencies like WHO, UNAIDS and NIH. Research scientists involved in SMC studies indicated that these global agencies were the primary policy audiences and attracted more direct engagement and dissemination of evidence. SMC research in Uganda, Kenya and South Africa was funded by these global stakeholders, who were also influential for trans-national policy diffusion.

Most importantly, the financing of SMC intervention was also perceived to arise from global or Western fund holders like the Global Fund and USA’s Presidential Emergency Fund for AIDS Relief (PEPFAR). Ugandan (and other) national level stakeholders were therefore treated as secondary audience by SMC research scientists –due also to the overall architecture of multi-country studies which require a global-structure that collates evidence from different countries and provides technical policy recommendations to all HIV affected countries. WHO and UNAIDS act as global-structures for SMC research and developed policy briefs and provided technical advice to all countries to add SMC to their HIV preventions strategies. One of the researchers vividly explained:

“the male circumcision studies were funded by NIH and WHO. We were called to Geneva to join research teams from Kenya and South Africa to guide the policy development by WHO and UNAIDS. […] we also developed a dissemination strategy for the results and engaged the (Ugandan) Ministry of Health. […] but let us face the facts - the Ministry of Health will not proceed with male circumcision without the funds from PEPFAR or Global Fund. So our dissemination in Geneva had all these agencies represented.” (Research Scientists).

#### Demand for feasibility research

At the time of this research, a Ugandan national SMC taskforce had been formed comprising MOH officials, and surgeons to advise about the implementation of the program. A striking similarity with PMTCT policy evolution was the demand for a series of additional research studies regarding feasibility of SMC implementation. As the Ministry of Health started to discuss the translation of the SMC evidence into policy, feasibility questions emerged that demanded additional operations research. The text below from a policy maker highlights this view:

”The issue of feasibility is very important. SMC should be simple and user friendly to our health providers because they are the ones who are going to implement it. [...] at the end of the day we are going to choose what is most user friendly to health care providers. Secondly, is the issue of acceptability to the general public. We are also looking at cultural sensitivity. We have been implementing HIV prevention strategies already - the ABC +. So the way we are introducing circumcision must not kill the good things which are already in HIV prevention. So those are some of the considerations to make sure that our SMC policy becomes more feasible and more acceptable”(Policy Maker).

With the support of donor agencies like Family Health International and Futures Group, studies were commissioned to assess the SMC acceptability, system-level readiness, and the potential cost-effectiveness of the strategy. A pilot for SMC was set up in one district in Uganda – Kayunga - to generate more operational evidence for policy learning and translation. From this perspective, the strong proof-of-concept evidence from clinical trials was not adequate for the decision makers. The demand for additional evidence suggests that a broader package of evidence is needed for the policy process to advance. The evidence package needs to incorporate feasibility assessment of adopting the new interventions.

*“I think the Ministry of Health tries to do its part but many of the interventions from research are not cheap and are not easy to implement*, *the challenge of human resource*, *financial resources and also the whole issue of scale-up. We can implement something in a small unit but to implement it across the whole country does require a lot more manpower and commitment from higher power beyond the Ministry”. (Research Scientist)*

#### Less visible evidence for SMC

Unlike PMTCT, the benefits for SMC are said to accrue after a large number of the eligible population has benefited and these benefits are often less tangible for policymakers. Numbers and rates association with SMC were less well known and more challenging to understand. Communication and decision-making tools such as mathematical modeling were being used to provide visual aids for future impacts and benefits in HIV prevention by SMC investments. One of the respondents among the researcher scientist expressed this as a complex process:

*”First of all you need to do a needs assessment*, *you need to do a mapping of the Health facility whether they exist or are able to shoulder circumcision. You need to come up with a communication strategy*, *you need to do a modeling of the costs and impact of circumcision on the HIV epidemic in Uganda. All these are needed to support implementation of circumcision.” (Research Scientist-)*

### What evidence should drive policy?

Respondents were asked about the preferred characteristics and sources of evidence and their motivation and incentives for generating, disseminating or utilizing health related research findings. Table 4 summarizes the nature and types of responses and reflects the contrasts in values, rationale for sharing, use and nature of evidence by the three groups of stakeholders. The data is collated under three themes ‘Incentives and values’, ‘rationale for communication’ and ‘strength of evidence’.

**Table 4:** Preferred characteristics and sources of evidence for policy in Uganda.

Concepts assessed	Researchers	Decision makers /funders	Media and CSO
Incentives and values for research generation or utilization	- Contribution to science and public health - Prospects for career development - networking with other experts - Income generation	- Optimizing wellbeing of communities - Demonstrating results & visible benefits - Improving value for money –(cheaper and effective) - Simplifying interventions	- Duty to inform the public - Grip and sustain audiences’ attention; - Generate revenue or goodwill - Promote debate with different view-points

Interests during dissemination or communication of evidence	- Focus is positive results, i.e. what worked well - Hands-off: “A good study speaks for its self” - Hand-over: give report to decision makers - Involve stakeholders in research processes	- Feasibility of applying new evidence: - how to achieve the benefits in the real world context - How much will it cost to implement? - How to integrated evidence with on-going policy and practice?	- Focus is to spur individual-level actions/awareness of the audiences - Simplify information for non-educated audience - Prefer known experts as the source of evidence - Value evidence from first-hand face-linked (experiential) evidence in addition to face-free sources

Criteria for judging strength of evidence	- Cohort studies, Randomized studies - Optimal representativeness – i.e. multi-site or multi-country studies - Statistical credibility - Long follow-up period of cohorts	- Operations research – testing the feasibility space eg benefits, acceptability, cost-effectiveness, and how to implement new interventions - Alignment of evidence to major operational challenges - Timeliness for action - Large effect size - Indigenous evidence - Reputation of research institution /researcher	- Aim is a “balanced” story – with “triangulation” of different perspectives e.g. proponents, opponents, service providers, decision-makers - Newsworthiness of evidence - Number of people affected - Extent of changes from status quo - Credibility of experts /researcher - Verification of evidence - Stories of affected people - Publications in major journals and conferences

CS: Civil Society Organizations;

#### Incentives and values for communicating and sharing research results

Our data revealed some differences in the values prevailing amongst researchers, decision makers and media practitioners. The contribution to science, career development, and being part of a large scientific network, were some of the incentives for researchers to participate in research. The attraction of decision makers to research was mostly driven by the need to find simpler and cost-effective solutions that address the major bottlenecks for improving the welfare of their communities. Media practitioners were mostly motivated by the duty to inform the public, entertain and generate debate, and maintain the interest of the audience while generating income and goodwill from the public. The need to generate debate was also important – usually implying that different views, some contrary to researched evidence are encouraged by media practitioners.

"Generating debate on major issues is the main incentive. One of the stories which really gave me international recognition, was this argument between the Global Fund, Ministry of Health and Ministry of Finance. The Global Fund’s view was that its money should just be additional, - to increase health spending. The Ministry of Finance was saying that increasing health spending beyond a certain fixed ceiling will cause economic problems for the country. It was a very interesting debate” (Media Practitioner-Print)

Our results show resistance to uptake of SMC due to the nature of the intervention, as well as the underlying values espoused by high level decision makers in the government. Research narratives show instances, where high level decision makers publicly expressed views doubting the evidence of SMC by citing popular observations of HIV infection among circumcised individuals and communities. They also worried about the misunderstanding or impact that SMC could cause to other interventions like condom distribution. Our findings show that once high level policy makers had publicly expressed unsupportive views about the SMC evidence, other decision makers in the lower ranks were uncomfortable to contradict high authorities. In their narrative, lower ranking policy makers worried that their job security would suffer if they in turn championed the policy process. In this circumstance, the media and civil society in general, assumed the role of championing the SMC process.

“Research shows that SMC has 60 percent protection but the problem is that while other governments have gone ahead, Uganda is not moving. Government is taking its time over policy. It is not a priority, the leadership is not convinced. Some top leaders say it does not work, the ministry is saying go and popularize circumcision. So there are contradictions but we try to advocate for it (Media Practitioner – Print)

#### Rationale for communication among the three groups

The main rationale for researcher-policy maker communication was driven by the need to share positive findings from research. Many researchers’ attitudes were not favorable to active engagement in dissemination of their findings to policy makers or the public. Instead, passive approaches e.g. sharing of reports with decision makers or media was preferable. Active approaches like face-to-face meetings, on-going dissemination and advocacy were considered as time wasting as one of the researchers referred to it as– “dancing in the corridors”. Hands-off approaches such as downloading journal publications by policy makers were considered sufficient by many research scientists. In contrast, decision makers were interested in research findings that advanced their contemporary problems in expanding welfare programs. Operations research findings that provided answers to challenges like how to finance, implement and scale-up programs like PMTCT and SMC were preferred.

Media practitioners reported that they preferred evidence from a locally recognized expert as opposed to a journal. Their interest was to access “famous” experts that would “put a face or voice” to the evidence. In this respect, the media usually seek senior research scientists although with little success and lots of frustration. Most media practitioners complained that research scientists have no time for them and use scientific jargon to communicate.

”I really would prefer to speak to the medical professionals unfortunately they tend to be press shy, generally hard to catch…….. the majority think that giving information to the press is a waste of their time. They don’t trust the press. Even when they have goodwill they use a very difficult language. The journalists get terrorized by the medical jargon” (Media Practitioner-Print)

#### Criteria for judging strength of Evidence

This study revealed that the three groups of stakeholders in this study expressed marked differences in the way they judge evidence to be useful for decision making, amplification, and dissemination. The research scientists assigned high weights to evidence that comes from cohort studies, randomized controlled trials , and multi-site studies to assure representativeness.

“We want randomized controlled trials but some of the observational cohort- especially the large cohorts conducted over a long period of time- can give good information on how best to manage certain conditions” (Research Scientist).

Decision makers assigned more weight to research that addresses operational problems they face at the time, and did not care much about the design of the study but focused on its relevance to the problem at hand. Overall, decision makers expected a broader set of evidence especially for new innovations like SMC. They expressed demand for evidence to address the technical, political, economic and social-cultural dimension of new interventions like SMC. At the time of this research, only the technical dimension of SMC was available from the randomized controlled trials [18,29,30].

For media practitioners, the importance of evidence was driven by the need to have several views represented to achieve a “balanced” story as well as good timing. In this process the media assigned high weights to experts who would be available to talk about the evidence and to the evidence that addressed a “hot” issue. For television, a widely known expert was vital to building credibility for the story or evidence. The quote below highlights how a triangulation of sources of evidence was needed for a credible story to be telecast:

“We use several sources in one story. One source is experience. Someone has been affected by a problem he is an authority. We have experts. By experts I mean people who are qualified in that specific area like a doctor, a gynecologist […]. Then we have researchers with new findings and publications. The other source is the policy makers or policy implementers. We also seek people in the system; we have to ask them – we have to confirm certain issues with them. People with different views are also important. A good story is one that brings out the different points of view.” (Media Practitioner – Television)

On the other hand, the print media practitioners looked at the story’s impact, how topical the story was, and the multiplier effect – how many people will read the story and how many will it inform - as critical to their analysis.

”There is the currency, the impact, the prominence, but also what is called the surprise element – something unusual, for instance a group of scientists came up with evidence that some people appear to be resistant to HIV. Too many people, that was the unusual surprise and you can’t believe, that day, the newspaper almost sold-out because of the headline. People wanted to find out more. How is it possible that someone can be resistant to HIV? I think those are the leading considerations for a good story”. (Media Practitioner – Print)

Media practitioners emphasized the number of the population affected, extraordinary evidence, and the need to create attention – sometime in a manner that may exaggerate the evidence. The practices of media practitioners include extraction of information from journals especially those that are well known – although this approach is usually supplemented with local experts. These findings provide opportunity for the experts in universities to become credible sources of information for the media. Nonetheless, there are significant challenges for these experts at universities and similar institutions to effectively play this role. Findings in this research revealed significant mistrust, unhelpful attitudes, and institutional barriers for researchers to engage with the media practitioners and advocacy agencies in general.

## Discussion

Findings from this study call for greater engagement of research generating institutions such as MakCHS and other academic institutions or agencies influential in health policy development and policy implementation. As detailed below, research and training institutions like MakCHS need to develop strategic competencies to generate, assemble and disseminate evidence that address the major feasibility constraints in implementing health interventions and innovations if these innovations are to become institutionalized into health policies and practices.

This study demonstrates that PMTCT policy uptake and continued use of implementation research to improve programs was possible due to a shared platform for learning and decision making across various stakeholders in Uganda. In the early phase of the PMTCT policy development, feasibility studies were undertaken to provide additional evidence needed for policy scale-up. In addition, positive program benefits were visible to policy makers and these benefits were being used as tools for advocacy in resource mobilization for scale-up [31,32]. The feasibility of implementation of the PMTCT policy was markedly enhanced by the building of a coalition of stakeholders – inclusive of researchers, funders, technical agencies and government officials. Although external funds were used to implement PMTCT programs, there was a national body assembled that included all major stakeholders. Evidence from policy development elsewhere shows that coalitions are useful in sharing information, resources and experiences that enable consensus on policy making and ease the feasibility of implementation [31,33].

Getting to a policy though important, does not guarantee that policy benefits will get to the communities or beneficiaries. For PMTCT, additional operational research was central in guiding and improving the implementation of the policy in Uganda. Even before the PMTCT policy was passed, implementation challenges guided the decisions towards the policy development processes. Findings for SMC show a similar pattern where additional evidence was required about implementation issues *before* the policy was passed. In view of these findings, it is imperative that implementation issues ought to be explored up-front to ensure that innovation-type research like PMTCT and SMC move forward into policy and programs [34]. The two case studies illustrate the influence of external stakeholders especially willing to finance the implementation of the programs thus boosting the policy development processes.

Lessons from SMC program in Uganda suggest that despite two years of vigorous national and international dissemination of the SMC research evidence, the policy process had stalled. The dissemination of evidence around SMC prioritized global agencies relative to national ones, and national level stakeholders were treated as secondary audiences; this might be one reason for the slow uptake of evidence. Key to the process was the lack of easily visible “benefits” which policymakers could see. Although averting HIV infection is a laudable policy goal, the benefits are invisible, and take decades to realize. In addition policymakers contested the SMC research evidence mostly due to concerns such as political feasibility, cultural values and discomfort with complex messages. This made the research to policy process much slower and more complicated in the case of SMC but reflects the fact that the content of the policy and the context influence the pace of evidence utilization in policy development.

This study also suggests that one of the main rationales for researcher-policy maker interaction was driven by the need to share positive findings. These findings demand an active engagement of researchers, policy makers and media practitioners to ensure effective dissemination of the research; Engagements such as deploying knowledge brokers, have been recommended in the literature to bridge the gap among these key stakeholders [35]. Divergent view on what type of evidence should drive policies showed that incentives, values and rationale of different stakeholders are important in Uganda. Our findings show that the nature of evidence was weighed differently by stakeholders; for instance, researchers preferred strong methodologies for policy evidence while policymakers looked at feasibility of implementation and also expected a broader set of evidence especially for innovations like SMC. Decision makers in Uganda needed evidence that covered all the major dimensions of implementation feasibility like social acceptability, cost-effectiveness, community benefits and health system readiness to deliver the intervention. For the media, the news value of a story, good timing and opinions from experts – professionals, affected persons and research scientists – were critical. Strategies need to be instituted to overcome barriers that media practitioners face to access the research evidence and experts. Institutional incentives, support mechanisms like training programs need to be designed to encourage a culture conducive to active interpersonal engagement among the three groups of stakeholders – researchers, Policymakers and media practitioners.

Researchers need to understand the limitations of their evidence as well as the motivation of other key stakeholders. Researchers in Uganda and in similar contexts also need to choose their media strategy – to inform policy makers, educate communities, or contribute to media debate to achieve progress in the health policy processes. Health training institutions like Makerere University College of Health Sciences may need to cultivate coalitions and inter-institutional linkages that increase joint and shared learning with key institutions that influence health decision making. In the same vein, institutions of higher learning need to raise the public profile of key experts and support them to undertake media relations work. Building credibility need to go hand in hand with expanding the engagement of these institutions in operations research that responds directly and in a timely manner to the concerns of policy makers especially for high stake health policies and programs.

### Implications for policy and practice

The implications of these findings for Uganda (and to the extent they resonate for other African nations) are four-fold: 1) Although the scientists would prefer rigor and take their time in the generation of credible evidence, policy makers are more concerned about the local relevance and “indigenization” of research evidence – and they are also concerned with visible effects accruing from applying the research evidence. 2) Where feasible, researchers may need to build into their studies clear measures of success in implementing their recommendations and means of demonstrating the changes attributable to their interventions. Where this is not feasible researchers will need to find ways to portray future benefits along with the feasibility of implementation. Mathematical modeling was found to be useful in the case of SMC [36]. 3) Media engagement would be enhanced if researchers invited to engage with the media were exposed to the research process earlier and through managed interactions. Researchers can focus on the public benefits of their work and where appropriate, video documentaries and testimonies can be useful for media-based communication of evidence. And finally, (4) researchers need to prepare adequately for dissemination of controversial findings – such as providing alternative explanations and limitations of their findings, methods and contexts. Institutional mechanisms for managing conflict of interests in the research processes need to be credible and functional to mitigate the controversies that could be exposed by the media’s approach to constructing newsworthy stories. Sensational reporting of scientific results by the media need to be born in mind when researchers are disseminating their findings. Effective communication of the implications and limitations of the research findings are needed to avoid erroneous extrapolations and exaggerations.

Academic institutions in Uganda and in similar context elsewhere ought to frame evidence in the formats used by media and decision makers. For media-oriented dissemination, there is need to format the evidence to reflect the implication of the evidence to individuals affected, potential implications to the person, community and population. For policy makers, the evidence needs to address feasibility constraints such as money, technology and acceptability of evidence-based innovations by the beneficiaries. Where funds for policy implementation are externally sourced, an assessment of policy feasibility need to include this dimension. It is also vital that an assessment of the techno-political benefits and risks of the innovations are addressed if the evidence-based innovations are to overcome the political economy litmus test. Overall, this study shows that effective translation of research might demand a “360 degree” approach to assembling the scope and nature of evidence needed to inform policy processes. Academic institutions also need to rethink their training of researchers to expand competencies in the presentation of information in multiple formats preferred by both media and decision makers. In the same vein, the media and decision-makers ought to develop capability to engage with the evidence in the formats used by the scientific communities. Provision of incentives within institutions – media, academic and decision making bodies – to value and prioritize activities for networking and sharing information would be helpful. Among the decision making institutions, an enabling environment that supports individuals to champion evidence-based changes with less risks to their careers would be ideal.

## List of abbreviations used

MakCHS: Makerere University College of Health Sciences; PMTCT: prevention of mother-to-child transmission; SMC: safe male circumcision; MDG: Millennium Development Goal; HSSP: Health Sector Strategic Plan; WHO: World Health Organization; LMIC: low and middle income countries; ART: antiretroviral therapy; IDI: in-depth interview; MOH: Ministry of Health; PEPFAR: Presidential Emergency Fund for AIDS Relief; COHRED: Council on Health Research for Development.

## Competing interests

The authors declare that they have no competing interests.

## Authors' contributions

All of the authors were involved in the conceptualization, tool development, analysis of the data, and writing of the manuscript. FS, LA, SNK, PP, NG were involved in the data collection. FS and LA led the analysis and preparation of the first draft of the manuscript. All authors revised drafts, read and approved the final manuscript.
